# Toll-Like Receptor-4 Coordinates the Innate Immune Response of the Kidney to Renal Ischemia/Reperfusion Injury

**DOI:** 10.1371/journal.pone.0003596

**Published:** 2008-10-31

**Authors:** Wilco P. Pulskens, Gwendoline J. Teske, Loes M. Butter, Joris J. Roelofs, Tom van der Poll, Sandrine Florquin, Jaklien C. Leemans

**Affiliations:** 1 Department of Pathology, Academic Medical Center, University of Amsterdam, Amsterdam, The Netherlands; 2 Center for Experimental and Molecular Medicine, Academic Medical Center, University of Amsterdam, Amsterdam, The Netherlands; Centre de Recherche Public-Santé, Luxembourg

## Abstract

Toll-like receptors (TLRs) can detect endogenous danger molecules released upon tissue injury resulting in the induction of a proinflammatory response. One of the TLR family members, TLR4, is constitutively expressed at RNA level on renal epithelium and this expression is enhanced upon renal ischemia/reperfusion (I/R) injury. The functional relevance of this organ-specific upregulation remains however unknown. We therefore investigated the specific role of TLR4 and the relative contribution of its two downstream signaling cascades, the MyD88-dependent and TRIF-dependent cascades in renal damage by using TLR4−/−, MyD88−/− and TRIF-mutant mice that were subjected to renal ischemia/reperfusion injury. Our results show that TLR4 initiates an exaggerated proinflammatory response upon I/R injury, as reflected by lower levels of chemokines and infiltrating granulocytes, less renal damage and a more preserved renal function in TLR4−/− mice as compared to wild type mice. *In vitro* studies demonstrate that renal tubular epithelial cells can coordinate an immune response to ischemic injury in a TLR4-dependent manner. *In vivo* we found that epithelial- and leukocyte-associated functional TLR4 contribute in a similar proportion to renal dysfunction and injury as assessed by bone marrow chimeric mice. Surprisingly, no significant differences were found in renal function and inflammation in MyD88−/− and TRIF-mutant mice compared with their wild types, suggesting that selective targeting of TLR4 directly may be more effective for the development of therapeutic tools to prevent I/R injury than targeting the intracellular pathways used by TLR4. In conclusion, we identified TLR4 as a cellular sentinel for acute renal damage that subsequently controls the induction of an innate immune response.

## Introduction

Inflammation at the site of tissue injury is a hallmark of almost all forms of renal injury and is an important factor in the development of many kidney diseases. Inflammatory cells can either mediate the initiation and progression of damage by direct cytotoxicity, secretion of soluble factors and regulation of immune responses, or can promote tissue repair and remodeling by production of growth factors and clearance of injured cells. It has become clear that renal epithelium plays a crucial role in the attraction of leukocytes upon injury [1], at least partially in a Toll-like receptor (TLR)-dependent manner [2].

The family of TLRs consists of highly conserved pattern recognition receptors that detect specific pathogen-associated molecular patterns such as peptidoglycan (TLR2) or lipopolysaccharide (TLR4) [3]. Interestingly, TLRs also recognize specific endogenous danger molecules that have been altered from their native state or accumulate in non-physiologic sites or amounts during tissue injury, such as heat-shock proteins, hyaluronan, high-mobility group box 1 protein (HMGB1) and fibrinogen [4]. Upon ligand recognition, TLRs are activated and initiate a proinflammatory response by the release of cytokines/chemokines and attraction of inflammatory cells [5], [6]. Except for TLR3, all TLRs control these innate immune responses through a conserved downstream signaling pathway, starting with the translocation of the adapter molecule MyD88 (myeloid differentiation factor 88) that ultimately leads to the early activation of NFκB [7]. Besides this pathway, TLR4 and TLR3 can use an alternative signaling cascade, the MyD88-independent route [8], which specifically involves the translocation of adapter molecule TRIF (TIR domain –containing adapter inducing IFN-β) [9], [10], in combination with the adapter protein TRAM (TRIF-related adapter molecule) that subsequently leads to the production of IFN- β and the expression of Interferon β-inducible genes [11], [12]. The diversity and specificity of the function of TLRs is determined by the selective use of these intracellular adapter molecules.

Where it was first thought that TLRs were mainly expressed on antigen-presenting cells, recent observations demonstrate that TLR mRNA expression is also present within solid organs such as the heart, liver and kidney [13]. In the kidney, the majority of the constitutive TLR2 and TLR4 mRNA is expressed by tubular epithelial cells (TECs) and is enhanced upon renal ischemia/reperfusion (I/R) injury as shown by *in situ* hybridization [14]. Importantly, the endogenous ligands that can activate TLR2 and TLR4 are strongly upregulated in these TECs upon I/R injury [15]. Together, these data suggest a potential role for renal TLR2 and TLR4 in the primary mechanism through which the kidney monitors renal injury and initiates and regulates inflammation. Indeed, we already demonstrated that renal-associated TLR2 plays a proinflammatory and subsequent detrimental role during I/R injury in the kidney of mice [2]. TLR4 can however exert different immunological effects as demonstrated by studies showing diverse effects of TLR2 and TLR4 in infection [16], [17], [18] and tissue injury models [19], [20]. This could be due to the fact that TLR4 detects other (endogenous danger) ligands, can signal via an alternative signaling cascade and does not hybridize with other TLRs as TLR2 does. The particular role of TLR4 in I/R injury remains therefore unknown. The definition of the specific roles of the MyD88-dependent and –independent pathways in TLR signaling might offer new possibilities for the selective blockade of pathways downstream of TLRs. Together, this prompted us to investigate the role of TLR4 and the relative contribution of the two individual downstream signaling cascades of TLR4 in I/R injury and repair in the kidney.

## Results

### Preserved renal function in TLR4−/− mice after I/R induction

**Figure 1:**
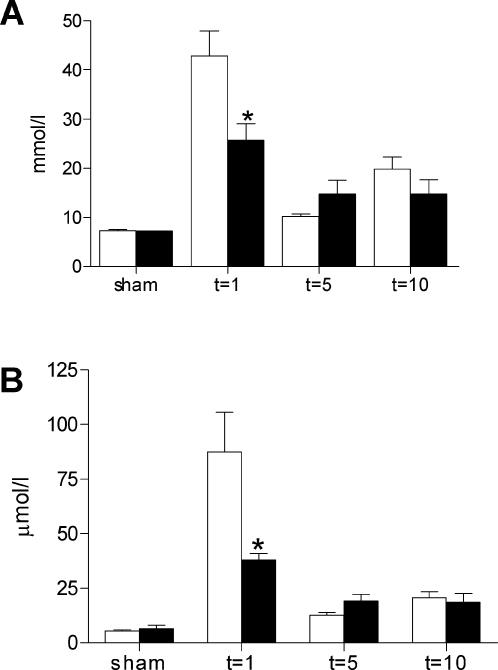
Renal function parameters of wild type and TLR4−/− mice. Renal function of TLR4−/− mice (black bars) was improved compared with that of the wild type mice (white bars) one day after renal I/R as reflected by lower serum urea (A) and creatinine (B) levels. Data are mean±SEM of 8 mice per group (sham-operated animals: n = 3/group). * p<0.05.

To evaluate the role of TLR4 in renal I/R injury, plasma urea and creatinine levels were determined in TLR4−/− and wild type mice. As shown in figure 1, I/R injury caused a transient increase in urea and creatinine levels in wild type mice, demonstrating a significant degree of renal dysfunction. Most importantly however, TLR4−/− mice were partially protected against renal dysfunction as reflected by significantly lower levels of plasma urea and creatinine levels compared with wild type mice one day after I/R injury. Five and ten days after I/R injury urea and creatinine levels went almost back to sham levels, and no differences were observed between TLR4−/− and wild types.

### TLR4 deficiency diminishes tubular injury

**Figure 2:**
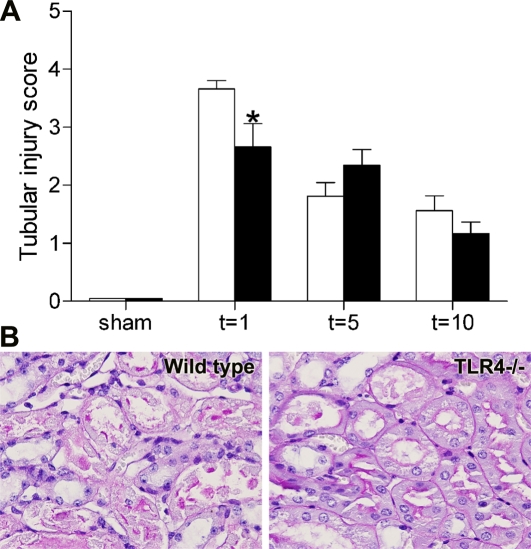
Scoring renal tubular damage of wild type and TLR4−/− mice. Score for histopathology after renal I/R injury (A) using PAS-D-stained renal tissue sections (B; representative for t = 1). Tubular damage was significantly lower in the outer medulla of kidneys of TLR4−/− mice (black bars) than in kidneys of wild type mice (white bars) one day after I/R injury. Data are mean±SEM of 8 mice per group (sham-operated animals: n = 3/group). * p<0.05.

In line with the preserved renal function in TLR4−/− mice, kidneys from these animals showed significantly less renal tubular damage such as widespread tubular necrosis, tubular dilatation, brush border loss and cast formation in the outer medulla one day after I/R injury compared with wild type kidneys (figure 2). Again, no significant differences were found in renal damage five and ten days after I/R injury between the TLR4−/− and wild type kidneys when renal function was already restored.

### Reduced influx of inflammatory cells in TLR4−/− kidneys

**Figure 3:**
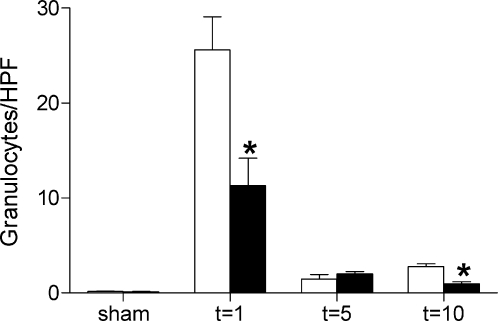
Renal influx of granulocytes in wild type and TLR4−/− mice. Influx of granulocytes in kidneys from wild type (white bars) and TLR4−/− (black bars) kidneys 1, 5 and 10 days after renal I/R injury or sham operation. One and ten days after I/R injury the number of granulocytes was significantly lower in kidneys of TLR4−/− mice than in kidneys of wild type mice as counted in 10 randomly selected high-power fields (HPFs) on outer medulla (magnification ×400). The amount of granulocytes from 8 mice per group were counted on renal tissue sections stained for Ly-6G and presented as mean±SEM. * p<0.05.

In order to obtain insight into the role of TLR4 in renal inflammation, we compared granulocyte and macrophage influx in kidneys of TLR4−/− and wild type mice by immunohistochemistry. Kidneys of wild type mice showed a transient increase in granulocyte influx with a peak one day after I/R injury (figure 3). Interestingly, TLR4−/− kidneys showed significantly lower amounts of infiltrated granulocytes one and ten days after I/R injury compared with wild type kidneys. Analysis of macrophage influx revealed that there was an increased infiltration of these cells in the corticomedullary region with a peak ten days after I/R injury in kidneys which was similar in both TLR4−/− and wild type mice (3.020±0.862 vs. 3.240±0.600 percentage staining/HPF).

### Reduced amount of cytokines and chemokines in TLR4−/− mice

**Figure 4:**
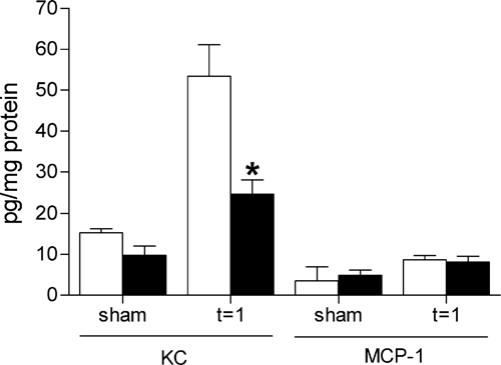
Levels of proinflammatory cytokines and chemokines in wild type and TLR4−/− mice. Expression of proinflammatory chemokines KC and MCP-1 in kidney homogenates of wild type (white bars) and TLR4−/− (black bars) mice one day after I/R injury or sham-operation. KC levels were significantly lower in homogenate of TLR4−/− kidneys compared with wild type kidneys, whereas there were no differences observed in MCP-1 levels. Data are mean±SEM of 8 mice per group measured in duplicate (sham-operated animals: n = 3/group). * p<0.05.

To evaluate whether early differences in the influx of granulocytes could be explained by differences in cytokine/chemokine production, we determined the concentrations of Keratinocyte Chemoattractant (KC) and Monocyte Chemoattractant-1 (MCP-1) in kidney homogenates of TLR4−/− and wild type mice one day after I/R injury. In accordance with the lower amount of granulocytes in the TLR4−/− kidneys, the levels of granulocyte chemoattractant KC were significantly reduced in the TLR4−/− kidney homogenates compared with that of wild type mice (figure 4). The levels of monocyte chemoattractant MCP-1 did not differ between kidney homogenates of TLR4−/− and wild type mice one day after I/R injury.

### Inflammatory response can be initiated by TECs via TLR4 in vitro

**Figure 5:**
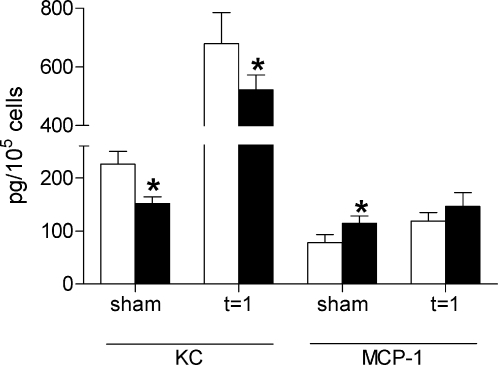
*In vitro* production of proinflammatory cytokines and chemokines by wild type and TLR4−/− TECs. Production of proinflammatory cytokines and chemokines by primary murine TECs from TLR4−/− (black) and wild type (white) mice, subjected to simulated ischemia. TLR4−/− TECs produce significantly lower amounts of KC as compared with TECs from wild type mice when subjected to simulated ischemia, whereas levels of MCP-1 were similar. Data are mean±SEM of 4–5 mice per group, measured in duplicate. * p<0.05. Ctr, control; Isch, Ischemia.

To evaluate whether renal epithelium can contribute to the initiation of an inflammatory response upon I/R injury in a TLR4-dependent manner, cultured primary tubular epithelial cells from TLR4−/− and wild type kidneys were subjected to simulated ischemia, and cytokine/chemokine levels were subsequently measured in the supernatant. As shown in figure 5, levels of KC were significantly lower whereas levels of MCP-1 were similar in the supernatant of TLR4−/− TECs compared with wild type TECs when subjected to simulated ischemia. The levels of TNF-α were below detection level. These data show that TLR4 on epithelial cells can indeed contribute to the induction of proinflammatory responses upon ischemic injury *in vitro*.

### Equal contribution of renal- and leukocyte- associated TLR4 to I/R injury in vivo

**Figure 6:**
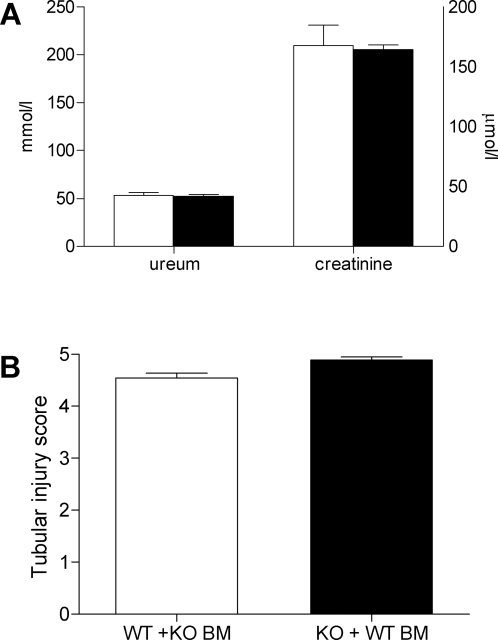
Equal contribution of epithelium-associated and leukocyte-associated TLR4 on renal function and injury one day after I/R injury. Renal function and injury of wild type mice reconstituted with TLR4−/− bone marrow (WT+KO BM, white bars, n = 7) was comparable to TLR4−/− mice reconstituted with wild type bone marrow (KO+WT BM, black bars, n = 9) one day after renal I/R injury, as reflected by equal levels of ureum (A left) and creatinine (A right) and tubular injury (B). Data are mean±SEM. * p<0.05.

In order to investigate whether the observed functional and morphological differences between wild type and TLR4−/− mice upon renal I/R injury could be ascribed to either renal epithelium-associated TLR4 or leukocyte-associated TLR4, bone marrow (BM) transplantation was performed to create chimeric mice. After seven weeks of engraftment, at least 80% of the leukocyte population of all mice consisted of donor-derived cells, indicating extensive engraftment (data not shown). These mice were subsequently subjected to severe renal I/R injury. It became clear that mice with renal-associated TLR4 (WT+KO BM) showed an equal degree of renal dysfunction compared to mice with leukocyte-associated TLR4 (KO+WT BM) one day after I/R injury as reflected by similar plasma levels of urea and creatinine (figure 6a). In addition, semi quantitative scoring of PASD-stained sections revealed that there was an equal level of renal injury between both groups (figure 6b). These results suggest that both renal-associated and leukocyte-associated TLR4 contribute to the observed functional and morphological differences that resulted from TLR4 deficiency upon renal I/R injury.

### Role of TLR4 in TEC apoptosis and proliferation after I/R injury

**Figure 7:**
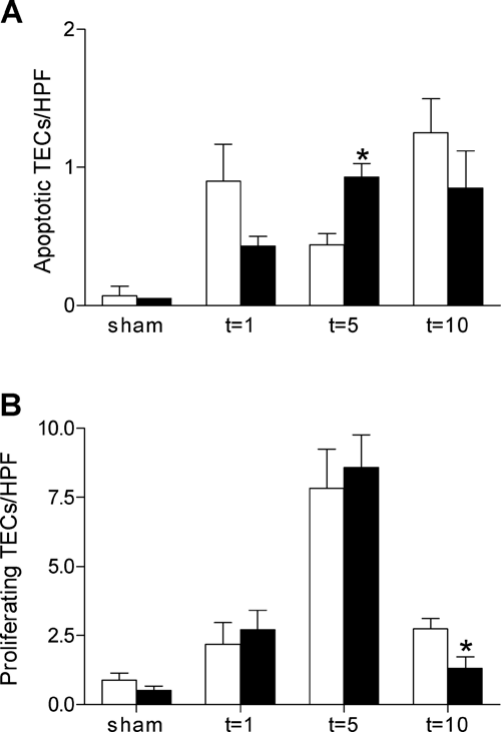
Apoptosis and proliferation of TECs of wild type and TLR4−/− mice. Apoptotic (A) and proliferating (B) tubular epithelial cells in kidneys from wild type (white bars) and TLR4−/− (black bars) mice at one, five and ten days after I/R injury or sham-operation. The amount of apoptotic tubular cells was significantly higher in the kidneys of TLR4−/− mice than in wild type mice five days after I/R injury, whereas ten days after I/R injury the amount of proliferating tubular cells was significantly lower in the kidneys of TLR4−/− than in kidneys of wild type mice as counted in 10 randomly selected non-overlapping high-power fields on outer medulla (magnification ×400). Positive tubular epithelial cells from 8 mice per group were counted on renal tissue sections stained for active caspase-3 (apoptosis) and BrdU (proliferation). Data are presented as mean±SEM. * p<0.05.

To investigate if TLR4 subsequently plays a role in repair and regeneration processes after I/R injury, we determined the amount of apoptotic and proliferating tubular epithelial cells in kidneys from TLR4−/− and wild type mice. One and ten days after I/R injury, the number of apoptotic TECs tended to be lower in TLR4−/− kidneys compared to wild type kidneys (figure 7a). Surprisingly, five days after I/R injury, the amount of apoptotic cells was significantly higher in the TLR4−/− kidneys compared with wild type kidneys. Using a BrdU-immunostaining, a transient increase in the amount of proliferating tubular epithelial cells could be observed with a peak five days after I/R injury which was similar in both genotypes. However, ten days after I/R injury there was a significant lower amount of proliferating cells in the kidneys of TLR4−/− mice compared with the wild types (figure 7b).

### MyD88 or TRIF deficiency does not influence renal function and granulocyte influx after I/R induction

**Figure 8:**
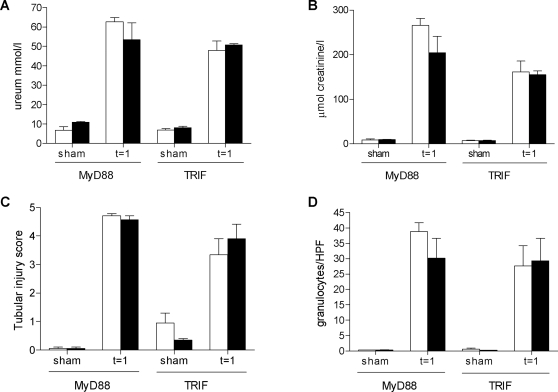
Renal function, injury and inflammatory influx in wild type, MyD88−/− and TRIF-mutant mice. Renal function of MyD88−/− and TRIF-mutant mice (black bars) did not differ compared with that of their wild type mice (white bars) one day after renal I/R as reflected by serum urea (A) and creatinine (B) levels and tubular injury (C). In addition, no differences were observed in the number of infiltrating granulocytes in kidneys of MyD88−/− and TRIF-mutant mice compared with their wild type mice (D). Data are mean±SEM of 6 (TRIF) or 8 (MyD88) mice per group (sham-operated animals: n = 2/group (TRIF) or n = 3/group (MyD88)). * p<0.05.

After having established that TLR4 plays a key role in initiating a proinflammatory response affecting renal function after I/R induction, we were interested in the relative contribution of the two separate downstream signaling cascades of TLR4 in renal I/R injury, one that relies on MyD88, and one that is mediated by TRIF. Since the differences in renal function, injury and granulocyte influx between TLR4−/− and wild type mice was most evident one day after I/R injury, further experiments were carried out at this time point. MyD88−/− mice showed a tendency towards decreased plasma urea and creatinine levels compared with wild type mice (figure 8a+b). However, these differences did not reach statistical significance. To verify these data, we performed an independent experiment with wild type and MyD88−/− mice and again did not find a significant difference in renal function (51.63±8.33 vs. 47.42±3.88 mmol/l ureum and 126.00±29.51 vs. 151.42±15.34 µmol/l creatinine) between both groups one day after I/R injury. The TRIF-mutant mice also did not show any differences in urea and creatinine levels compared with wild types (figure 8a+b). Moreover, no differences were observed in tubular damage between MyD88−/− and wild type mice (4.57±0.14 vs. 4.71±0.07) or TRIF-mutant and their wild type mice (3.90±0.52 vs. 3.34±0.56) after I/R injury (figure 8c). In line with renal function and histology, there were also no differences in granulocyte influx between MyD88−/− and TRIF-mutant mice compared with their wild types one day after I/R injury (figure 8d).

## Discussion

Upon renal I/R injury, inflammatory processes are abundant and contribute to the subsequent enhancement of tubular injury. Especially renal tubular epithelial cells are central players, since injury of these cells can result in the local synthesis of proinflammatory mediators and the subsequent attraction and migration of inflammatory cells into the injured renal parenchyma [1]. It is already demonstrated that tubular epithelial cells constitutively express TLR2 and TLR4, whose expression levels are upregulated upon I/R injury [14]. Whereas we have already shown that TLR2 plays a proinflammatory and subsequent detrimental role upon I/R injury *in vivo* contributing to renal dysfunction and injury [2], the functional relevance of increased TLR4 expression remains unknown. As TLR4 can recognize other endogenous ligands than TLR2 [21], can use an alternative signaling cascade [22] and does not hybridize with other TLR family members as TLR2 does, the functional significance of TLR4 during tissue injury could be different from TLR2. Indeed, different effects of TLR2 and TLR4 were found in both infection models [16], [17], [18] as in tissue injury models [19], [20]. Therefore, in this study we investigated the individual role of TLR4 upon renal I/R injury and demonstrated that TLR4 plays a proinflammatory role, as reflected by a reduced amount of infiltrating granulocytes and the chemokine KC in kidneys of TLR4−/− mice compared with wild type mice. Since an exaggerated inflammatory response could lead to more severe tissue damage, this enhanced inflammation could explain why TLR4−/− mice showed less tubular injury and a more preserved renal function compared with wild type mice upon I/R injury. These results are in line with studies describing an import role for TLR4 in lung and hepatic I/R injury models [23], [24], [20]. In addition, another study also reports that activation of TLR4 mediates renal I/R injury [25].

Whereas our *in vitro* data showed that primary murine TECs need TLR4 in order to produce significant amounts of cytokines/chemokines after simulated ischemia, the use of chimeric mice in a model of severe I/R injury revealed that there was a comparable relative contribution of renal-associated and leukocyte-associated TLR4 to the observed renal dysfunction and tubular injury *in vivo*. These results are partially in contrast to the report of Wu et al. which demonstrated that intrinsic renal cells play the dominant role in mediating kidney damage. A possible explanation for this discrepancy could be that the study of Wu et al. induced sublethal renal ischemia, whereas we subjected our chimeric mice to prolonged severe I/R injury. The abundant infiltration of inflammatory cells during lethal I/R injury thereby might overwhelm the primary response initiated by epithelium-derived TLR4. Interestingly, Cunningham et al. showed that extrarenal TLR4 rather than renal TLR4 mediated LPS-induced acute renal failure [26]. Although this model used a microbial ligand instead of endogenous stress molecules; it emphasizes the role extra-renal TLR4 can play in the kidney during disease. Moreover, Tsung et al. showed in a model of hepatic I/R injury that functional TLR4 signaling in non-parenchymal cells in particular is important for recognizing the initial damage from ischemic cells.

The diversity and specificity of a TLR function is determined by the selective use of the intracellular adapter molecules. The definition of the specific roles of the MyD88-dependent and TRIF–dependent pathways in TLR signaling might offer new possibilities for the selective blockade of pathways downstream of TLRs. We therefore investigated their relative contributions *in vivo*. This revealed that the TRIF-dependent cascade does not play an important role early after I/R injury, as reflected by a similar degree of renal dysfunction, tubular injury and granulocyte influx in the TRIF-mutant mice as compared with their wild types. That would point towards a more important role for the MyD88-dependent cascade in this model. Surprisingly however, we did not find differences in renal function, tubular damage and in numbers of infiltrating granulocytes between MyD88−/− and wild type mice in two independent experiments. In accordance with our observations, no important role for the TRIF-dependent pathway upon renal I/R injury was found in a study of Shigeoka et al. [27], whereas they did observe functional differences between wild type and MyD88−/− mice. MyD88−/−×TRIF−/− (double KO) mice were found to have a similar phenotype as the MyD88−/− mice, suggesting that the TRIF-dependent pathway is indeed not important.

From our data it became clear that the absence of TLR4 protected the kidneys to a higher degree that the absence of the downstream signaling molecule MyD88. Shigeoka et al. observed similar results showing that TLR2 deficiency provided more protection than a deficiency in MyD88 or TRIF individually or a combination of those two adapter molecules [27]. They suggested the possible existence of additional TLR2-dependent/MyD88-independent signaling pathways. Likewise, it seems reasonable that there are also yet unknown TLR4-dependent/MyD88-independent/TRIF-independent signaling pathways in this renal I/R injury model. Moreover, it is known that other receptors, including members of the Interleukin (IL)-1R/TLR superfamily such as IL1 and IL18, signal via the MyD88 adapter protein as well, and could thereby abolish the proinflammatory effects of TLR4 signaling [28], [29]. An alternative explanation is that endogenous ligands that are released upon I/R injury might activate other TLR-family members signaling via the MyD88-dependent cascade that could have an anti-inflammatory effect abolishing the proinflammatory signal of TLR4. Indeed, it is already demonstrated that TLR9 can trigger anti-inflammatory functions in experimental colitis [30], and it has been shown that upon TLR-signaling the levels of the anti-inflammatory cytokine IL-10 can be enhanced within dendritic cells [31]. Moreover, a study reported that individual molecules of signaling pathways can differentially regulate the TLR-mediated production of pro- and anti-inflammatory cytokines [32]. Recent reports even suggested the existence of non-TLR-dependent adapter molecules [33]. Obviously, further experimental work is necessary to elucidate the mechanisms through which TLR signaling leads to differential downstream responses.

Besides the initiation of a proinflammatory response, we also investigated if TLR4 activation was involved in the repair and regenerative processes that take place upon I/R injury, such as the clearance of damaged tubular epithelial cells by apoptosis and subsequent replacement of epithelium by proliferation of TECs. In contrast to the results we had already demonstrated for TLR2 [2], these findings suggest that TLR4 does not play a major role in the induction of apoptotic processes, despite a tendency towards less apoptotic TECs found in the TLR4−/− mice after one day of I/R injury. The latter would be in line with the less renal dysfunction and less inflammation that were observed at this time in TLR4 deficient mice, since apoptosis is thought to contribute to the initiation of reperfusion-induced inflammation and subsequent tissue injury [34]. Interestingly, despite similar levels of tubular injury five days after I/R injury, the amount of apoptotic cells was significantly higher in kidneys of TLR4−/− mice compared with wild type mice. This agrees with a previous study demonstrating that TLR4 signaling protects against apoptosis in the injured intestine [35]. Furthermore, in contrast to the effect what we already described for TLR2 deficiency [2], no effect of TLR4 deficiency was found on the amount of proliferating TECs on early time points, while after ten days of I/R injury the amount of proliferating TECs was lower in the TLR4−/− mice compared to wild type mice. This agrees with previous studies demonstrating that TLR4 signaling is required for proliferation in the injured intestine [35], and the demonstration that HSP60-mediated TLR4 activation stimulates vascular smooth muscle cell proliferation [36]. However, apoptosis and proliferation of TECs do not appear to contribute to the differences in renal function and morphology that were observed between wild type and TLR4−/− mice one day after renal I/R injury.

In conclusion, this study shows that TLR4 can initiate an exaggerated proinflammatory response early after renal I/R injury, contributing to renal injury and dysfunction, and indicates that both epithelium-associated and leukocyte-associated TLR4 contribute to the I/R induced effects *in vivo*. As deficiency of the intracellular adapter molecules MyD88 and TRIF did not influence the degree of renal injury and inflammation, selective targeting of TLR4 may be more effective for the development of therapeutic tools to prevent I/R injury than targeting the intracellular pathways used by TLR4. Moreover, these findings support the idea that the function of TLRs extends beyond host defense against invading pathogens; TLR4 seems to be a cellular sensor for acute renal damage that controls innate immunity and tissue integrity.

## Materials and Methods

### Mice

Pathogen-free 8–12 week-old male wild type (Wt) C57BL/6 mice were purchased from Charles River Laboratories. The TLR4−/−, MyD88−/− and TRIF-mutant mice, backcrossed six times to a C57Bl/6 background, were a kindly gift of the Center for Experimental and Molecular Medicine within the Academic Medical Center and bred in the animal facility of the Academic Medical Center in Amsterdam, The Netherlands. TLR4−/−, MyD88−/− and TRIF-mutant mice were originally generated as described previously [37], [38], [39], and the correct phenotype was revealed by genotypic screening. The CD45.1 positive allotype wild type mice used to create chimeric mice were purchased from Charles River Laboratories (B6.SJL-*Ptprc^a^Pepc^b^*/BoyCrl strain). Only age- and sex-matched mice were used in all experiments. The Animal Care and Use Committee of the University of Amsterdam approved all experiments.

### In vivo Ischemia/Reperfusion

Renal I/R injury was induced as described previously [40]. Briefly, both renal arteries were clamped for 45 minutes using micro aneurysm clamps through a midline abdominal incision under general anesthesia (0.07 ml/10 g mouse of fentanyl citrate fluanisone midazolam mixture containing: 1.25 mg/ml midazolam (Roche Diagnostics Corp.), 0.08 mg/ml fentanyl citrate, and 2.5 mg/ml fluanisone (Janssen Pharmaceutica)). After clamp removal, kidneys were inspected for restoration of blood flow. The abdomen was closed in 2 layers, and all mice received a subcutaneous injection of 50 µg/kg buprenorphin (Temgesic; Schering-Plough) for analgetic purposes and were allowed to recover from surgery for 12 hours at 28°C in a ventilated stove. To maintain fluid balance and volume status, mice were supplemented with a few drops sterile 0.9% NaCl intra-peritoneal. Sham-operated mice underwent the same procedure without clamping and were sacrificed one day after surgery. To mark proliferating cells, 5-bromo-2′-deoxyuridine (BrdU; Sigma-Aldrich) was injected intraperitoneally (30–50 mg/kg body wt) 60 minutes before sacrifice. Mice were sacrificed 1, 5, and 10 days after surgery. At these time points, mice were weighed; blood was collected by heart puncture in heparin-containing tubes and after 10 minutes centrifugation at 10,000 rpm stored at −80°C. Kidneys were snap-frozen in liquid nitrogen and stored in −80°C till further analysis.

### Plasma biochemical analysis

Plasma urea and creatinine levels were determined by routinely used clinical diagnostic urease and creatinase assays.

### Primary culture of mouse renal tubular epithelial cells

Kidneys from wild type (n = 4) and TLR4−/− (n = 5) mice were harvested and the capsule was removed. Tissue from the outer cortex was cut into pieces of approximately 1 mm^3^, and subsequently prepared by 1 mg/ml Collagenase type 1A (Sigma-Aldrich) dispersion at 37°C for 1 hour. After washing, primary TECs were grown to confluence on 6-well plates in HK2 culture medium supplemented with 10% FCS, 100 IU/ml penicillin, 100 µg/ml streptomycin, 2 mM L-glutamine (all from Invitrogen Corp.), 1% ITSe and 1% S1 hormone mixture (Sigma). The nature of primary cells was confirmed by culturing cells on glass slides that subsequently were stained for markers specific for different cell types. The majority of cells were positive for E-cadherin and ZO-1, whereas staining for α-SMA, F4/80 and CD68 remained negative (data not shown).

### In vitro simulated ischemia

Cultured primary TECs in 6-well plates were washed twice with PBS to remove waste products, and ischemia was simulated by immersing the cellular monolayer in mineral oil for 1 hour at 37°C as done before [2], [41]. Control cell monolayers remained in HK-2 medium. After extensive washing, cells were incubated for 24 hours in HK-2 medium. Supernatants were collected and stored at −80°C until ELISAs were performed (see below). The total amount of cells present in the wells after simulated ischemia was counted using a Coulter Counter (Beckman).

### Preparing kidney homogenate

For cytokine and chemokine measurements, snap-frozen kidneys were homogenized in Greenberger lysis buffer (containing 150 mM NaCl, 15 mM Tris, 1 mM MgCl·H_2_O, 1 mM CaCl_2_ and 1% Triton-X, combined with protease inhibitor cocktail (Sigma)), homogenized and incubated for 30 minutes at 4°C. The homogenates were subsequently centrifuged at 10,000 rpm for 10 minutes, after which the supernatants were stored at −80°C until ELISAs were performed. To determine protein content, the Bio-Rad Bradford Protein Assay (Bio-Rad laboratories) was used with IgG as standard.

### ELISA

Cytokine (TNF-α) and chemokines (KC and MCP-1) were measured using the specific ELISA kits (R&D Systems) according the manufactures protocol. The detection limits were 31 pg/ml for TNF- α, 13 pg/ml for KC and 16 pg/ml for MCP-1.

### Scoring of histopathology

All histopathological scorings were made in the cortex using PAS-D–stained renal tissue sections and performed on coded slides. The percentage of damaged tubules in the corticomedullary junction was estimated by a blinded pathologist using a 5-point scale according to the following criteria: tubular dilatation, cast deposition, brush border loss, and necrosis in 10 randomly chosen, non-overlapping fields (×400). Lesions were graded on a scale from 0 to 5: 0 = normal; 1 = mild, involvement of less than 10% of the cortex; 2 = moderate, involvement of 10 to 25% of the cortex; 3 = severe, involvement of 25 to 50% of the cortex; 4 = very severe, involvement of 50–75% of cortex; 5 = extensive damage, involvement of more than 75% of the cortex.

### Detection of granulocytes and macrophages

Renal tissues were fixed for 24 hours in 10% formalin and embedded in paraffin. Sections of 5 µm were deparaffinized and endogenous peroxidase was subsequently blocked using methanol with 0.3% H_2_O_2_. After digestion with a solution of 0.25% pepsin into 0,1 M HCl (Sigma-Aldrich) for granulocytes or with 0.1% Trypsin (BDH Chemicals) into PBS for macrophages, non-specific binding was blocked by incubation with 5% normal goat serum (Dako Cytomation) within phosphate buffered saline (PBS). Sections were exposed to FITC-labeled anti-mouse Ly6G mAb (BD Biosciences–Pharmingen) or rat anti-mouse F4/80 IgG2b mAb (Serotec) overnight at 4°C. For staining of granulocytes, slides were incubated with rabbit anti-FITC antibody (Dako Cytomation), which was followed by a further incubation with HRP-conjugated goat anti-rabbit IgG (Immunovision Technologies Co.). For macrophage staining, slides were incubated with rabbit anti-rat biotin (DakoCytomation), which was followed by streptavidin-ABC solution (DakoCytomation). The slides were developed using 1% H_2_O_2_ and DAB (Sigma-Aldrich) in 0.05 M Tris-HCl (pH 7.9) and counterstained with Methyl Green (Sigma-Aldrich). For granulocytes the number of positive cells was counted within 10 non-overlapping fields (×400). The amount of positive macrophage staining was quantified digitally with Image Pro Plus software version 5.0.

### Detection of apoptosis and proliferation

Tissue sections of kidneys were deparaffinized and boiled for 10 minutes in 10 mM Tris/1 mM EDTA (pH 9.0). Nonspecific binding and endogenous peroxidase activity were blocked as described above, followed by incubation with rabbit anti-human active caspase-3 polyclonal antibody (Cell Signaling Technology), followed by a further incubation with HRP-conjugated goat-anti rabbit IgG (ImmunoVision Technologies Co.), and further processed as described above. To stain for BrdU, DNA was denatured in 2 M HCl, and antigen retrieval was performed by 0.4% pepsin in 0.01 M HCl. Sections were then incubated in 5% normal goat serum, 0.1% bovine serum albumin (Sigma-Aldrich), and 0.1% Tween-20 (Sigma-Aldrich) and exposed to mouse IgG1 anti-BrdU antibodies (Sigma-Aldrich). This was followed by a further incubation with goat anti-mouse IgG1-HRP (SouthernBiotech). Finally, sections were developed as described above. The number of positive cells was counted within 10 non-overlapping fields (×400).

### Bone marrow transplantation

Total bone marrow was collected from TLR4−/− (C57Bl/6 background, CD45.2 alloantigen positive on circulating leukocytes) and wild type mice (alloantigen CD45.1 positive on circulating leukocytes (B6.SJL-*Ptprc^a^Pepc^b^*/BoyCrl), purchased from Charles River, Netherlands) [42] by flushing femurs and tibiae with sterile PBS containing 10% FCS, 100 IU/ml penicillin and 100 µg/ml streptomycin. To ensure short-term survival of the recipients, single cell suspension of a syngeneic spleen from a CD45.1 wild type or TLR4−/− mice was obtained by crushing spleens in PBS containing 10% FCS, penicillin and streptomycin through a 40-µm cell strainer (BD). Male TLR4−/− (n = 9) and wild type (n = 7) recipients were lethally irradiated with two doses of 5Gy, separated by 4 hours, using a ^137^Cs irradiator (CIS bio international; SCHERING S.A.). After the last irradiation dose, 5×10^6^ TLR4−/− or CD45.1 wild type bone marrow cells and 2×10^5^ TLR4−/− or CD45.1 wild type spleen cells in sterile PBS were injected into the tail vein of recipient irradiated mice. These mice were kept in micro isolator cages for 7 weeks to complete engraftment with donor bone marrow, after which renal I/R injury was induced as described above. One week prior and 5 weeks following transplantation, mice received sterile acidified tap water (12×10^−3^ M HCl) containing 0.16% neomycin sulphate (Sigma-Aldrich) to prevent the immunocompromised recipients from infections.

### FACS analysis

To assay bone marrow reconstitution, whole blood was collected from recipients when sacrificed and subsequently analyzed by flow cytometry to determine donor/recipient chimerism of the hematopoietic compartment. Erythrocytes were lysed for 10 minutes at 4°C in 160 mM NH_4_Cl, 10 mM KHCO_3_ and 0.1 mM EDTA (pH 7.4), and the remaining leukocytes were brought to a concentration of 4×10^6^ cells/ml FACS buffer (PBS supplemented with 0.5% BSA, 0.01% NaN_3_ and 100 mM EDTA) and stained for CD45.1 (clone A20, BD Biosciences) and CD45.2 (clone 104, BD Biosciences) for 30 minutes at 4°C. Chimerism was determined by analyzing the populations of CD45.1 and CD45.2 positive cells. To correct for background staining unlabeled cells were used. All analyses were performed using a FACSCalibur (BD) and WinMDI 2.5 software.

### Statistics

Differences between groups were analyzed using the independent samples T-Test. Values are expressed as mean±SEM. A value of P<0.05 was considered as statistically significant.
